# Impact of Synthesis Parameters of Multi-Walled Carbon Nanotubes on their Thermoelectric Properties

**DOI:** 10.3390/ma12213567

**Published:** 2019-10-30

**Authors:** Bogumiła Kumanek, Grzegorz Stando, Paweł S. Wróbel, Dawid Janas

**Affiliations:** 1 Department of Organic Chemistry, Bioorganic Chemistry and Biotechnology, Silesian University of Technology, B. Krzywoustego 4, 44-100 Gliwice, Poland; gstando13@gmail.com; 2 Centre of Polymer and Carbon Materials Polish Academy of Sciences, M. Curie-Sklodowskiej 34, 41-819 Zabrze, Poland; pwrbel06@gmail.com

**Keywords:** carbon nanotubes, thermoelectric properties, Seebeck coefficient

## Abstract

Carbon nanotubes have been intensively researched for many years because of a wide array of promising properties that they have. In this paper, we present the impact of synthesis parameters on thermoelectric properties of nanocarbon material. We conducted a number of syntheses of multi-walled carbon nanotubes (MWCNTs) at different temperatures (800 and 900 °C) using various amounts of catalyst (2%, 5.5%, and 9.6%) to facilitate the process. We also tested the influence of injection rate of precursor and the necessity of material purification on thermoelectric properties of MWCNTs. The electrical conductivity, thermal conductivity, and Seebeck coefficient were measurement for all samples. Based on these parameters, the values of Power Factor and Figure of Merit were calculated. The results show that the most important parameter in the context of thermoelectric properties is purity of employed MWCNTs. To obtain appropriate material for this purpose optimum synthesis temperature and appropriate content of the catalyst must be selected. The study also reveals that post-synthetic purification of nanocarbon is essential to produce an attractive material for thermoelectrics.

## 1. Introduction

The global consumption of energy has been rising year by year due to the ever-increasing development of our civilization. One of the key problems of this aspect of life, which results from low energy efficiency of devices, engines, and other appliances [1,2], is that a lot of waste heat is generated. The consequence is that we have to dissipate this heat by employing some sort of cooling such as heat sinks or heat exchangers, and this heat also makes a significant contribution toward global warming [3,4]. Self-consciousness and implementation of more and more stringent energy policies around the world regarding energy management gives strategic significance to the development of methods of harvesting and utilization of such energy. One of the methods to meet this challenge is to use so-called thermoelectric devices, which transform thermal to electrical energy on the basis of the Seebeck effect [5]. When exposed to the temperature gradient, certain materials are able to generate current, which can be further utilized for a variety of applications. Unfortunately, the materials with high efficiency of conversion of heat to electricity developed so far, are based on rare earth elements, which are not only difficult to obtain but can also be very expensive and toxic [2,6,7,8,9,10]. Examples of such materials include Bi_2_Te_3_, Sb_2_Te_3_, and PbTe. The end result is that thermoelectric devices are still not present in regular households but are rather used for specialized applications. The most notable is perhaps the power system of NASA’s Voyager 2 probe launched in 1977, which in 2018 left the Solar System. It is evident that it is necessary to search for the materials that both demonstrate high efficiency of energy recovery and are cheap to obtain to domesticate this technology. 

The materials that have significant potential for thermoelectric applications are carbon nanotubes (CNTs). The Seebeck coefficient at room temperature for individual semiconducting SWCNT was calculated by Hungh et al. [11], who reported that the Seebeck coefficient for this type material can exceed 2000 µV/K. However, as it is almost always the case, theoretical considerations provide higher values than those that we can observe empirically. The Seebeck coefficient at room temperature has been reported to reach the value of up to 200 µV/K for individual SWCNTs [12]. For networks constructed from such CNTs, the performance is between 80 µV/K and 160 µV/K (when the starting material is composed of semiconducting SWCNTs). Films made from mixed SWCNT have demonstrated values below 100 µV/K, and networks made entirely from metallic SWCNTs demonstrate values just on the order of 25 µV/K [13]. The ideal thermoelectric material has a semiconducting character, so such a trend is to be expected. As for MWCNTs, the literature includes information that value of the Seebeck coefficient for individual MWCNTs is about 80 µV/K [14], and for films made from them, approximately 20 µV/K [15] or less [16,17]. Jakubinek et al. [15] demonstrated that films made of vertically aligned arrays of MWCNTs are characterized by higher values of the Seebeck coefficient than that of isotropic films. Another example of thermoelectric performance of MWCNT films was shown by Kunadian et al. [18], who noted approximately −4.5 µV/K Seebeck coefficient at room temperature Lower values for MWCNTs in comparison with SWCNTs are associated with significant structural and electronic differences between these two CNT types. Plurality of walls in such materials play a negative role because some of these shells are of metallic chirality, which deteriorates the overall performance of the material.

A high value of the Seebeck coefficient is not the only property that a material must demonstrate in order to be potentially applied as a thermoelectric device. Other very significant characteristics are the values of electrical and thermal conductivity. Based on those parameters, the first index providing information on the ability of a material to recover energy and transform waste heat into electricity is a so-called Power Factor (PF, Equation (1)). It takes into the account the Seebeck coefficient and electrical conductivity, both of which should be as high as possible. On the other hand, when thermal conductivity is considered, the Figure of Merit (zT, Equation (2)) can be calculated. 

Power Factor (PF)

(1) $$PF=S^{2}\sigma$$

Figure of Merit (zT)

(2) $$zT=\frac{S^{2}\sigma }{\kappa }T$$

where S = Seebeck coefficient (V/K), σ = electrical conductivity (S/m), κ = thermal conductivity (W/mK], and T = measurement temperature (K).

Therefore, to obtain a material with good thermoelectric properties, it is necessary to find one characterized by a balance between the highest values of electrical conductivity and Seebeck coefficient while keeping the thermal conductivity to the minimum. So far, materials based on CNTs demonstrate zT values up to 0.42, depending on the doping agents used [5,19,20,21].

Despite their still relatively low-performance (as compared with solutions based on rare earth metals), the application of CNTs as thermoelectric materials for production of heat recovery devices is a promising route explored by many scientists around the world. Successful implementation of CNTs for this purpose would allow us to reduce costs because, upon application of the most common method of synthesis, i.e. Chemical Vapour Depositon (CVD) [22], they are much cheaper to produce than rare earth elements. CVD is an established industrial technology, and the process to make CNTs by this route is conceptually simple: a catalyst and a source of carbon are combined at high temperature under an appropriate gaseous environment. Since CNTs can be made from any feedstock containing carbon, these materials can also be obtained from renewable resources free of any geopolitical constraints (in contrast with the aforementioned rare earth metals). The key challenge at this point is to improve their thermoelectric performance. 

In our study, we wanted to find out how microstructure and chemical composition of CNTs affect their ability to generate electricity from waste heat. We conducted a series of syntheses of MWCNTs using the CVD method with various parameters: temperature, amount of catalyst and injection speed. Additionally, the catalyst was eliminated from one of the materials to verify its impact on thermoelectric performance. These experiments allowed us to understand how to tune the synthesis so as to make material with the highest potential for thermoelectrics.

## 2. Materials and Methods

**Figure 1:**
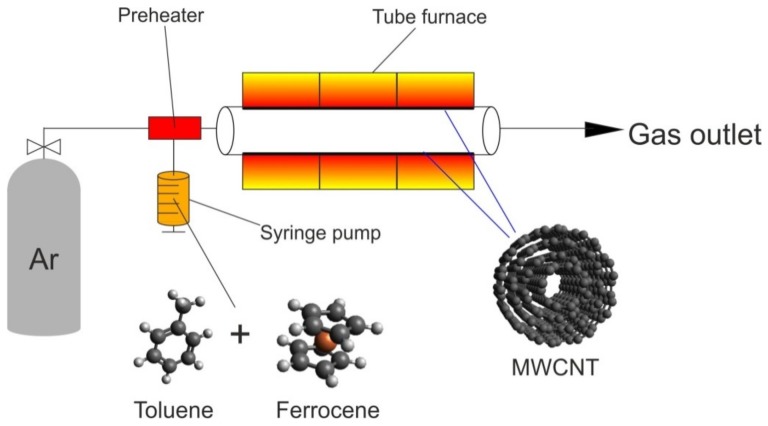
Scheme of multi-walled carbon nanotube (MWCNT) synthesis.

MWCNTs were synthesized by catalytic CVD [23]. The solution of ferrocene in toluene was dosed by a syringe pump (ESIRYR; New Era Pump Systems, NY, USA) into a tube furnace (Carbolite HST 12/900; Derbyshire, UK) kept in argon (Air Liquide; Cracow, Poland) atmosphere. The time of the process (four hours) was the same for all reactions. After finishing the injection, the furnace was cooled to room temperature to extract the product. Figure 1 shows the scheme of the synthesis setup.

A number of syntheses were conducted, in which various conditions were applied in order to determine their impact on the thermoelectric properties of MWCNTs (Table 1). MWCNTs were obtained using different temperatures (800 °C or 900 °C) and assorted amounts of the catalyst (2%, 5.5%, or 9.6%, calculated relative to the volume of toluene). Additionally, the impact of injection speed on the properties of obtained material was determined. The source of carbon in the synthesis was toluene (pure p.a.; Chempur, Piekary Slaskie, Poland) and it was facilitated by the presence of ferrocene (pure p.a.; Sigma Aldrich, Poznan, Poland) as a catalyst. 

Moreover, a selected sample of MWCNTs named 5.5%-5 mL/h-800 C was subject to purification for the purpose of validation of the impact of leftover catalyst from the synthesis on thermoelectric performance. The purification consisted of refluxing of 3 g of MWCNTs in 400 mL HCl (pure p.a.; Chempur, Piekary Slaskie, Poland) for 6 h. Then, the solution was diluted using distilled water to the volume of 1200 mL. Subsequently, the mixture was filtered through PTFE membrane (pore size: 0.22 µm; Fisherbrand, Ottawa, Canada) and washed, first by 5% NaHCO_3_ solution, and then by distilled water until reaching pH 7. The residue was dried in a dryer (115 °C) until obtaining stable mass. The purified film obtained this way was marked with the acronym 5.5%-5 mL/h-800 C-pure.

The names were assigned to samples based on the following principle: amount of catalyst-temperature of synthesis-injection speed. For example, 2%-5 mL/h-800 C means that MWCNTs were obtained with 2% content of catalyst at 800 °C, and the substrates were injected at the rate of 5 mL/h.

### 2.1. Preparation of MWCNT Films

Here, 90 × 90 mm films were obtained from the synthesized CNTs using the method reported by us earlier [24]. In short, first, equal weights (0.5 g) of MWCNTs and ethyl cellulose (EC) were combined in 80 mL of mixture of acetone-toluene (1:1, m/m). Appropriate dispersion of nanocarbon in the liquid medium was obtained using ultrasounds (time of sonication: 20 min, amplitude 100%, max. power 60 W). The homogenous mixture was then placed onto a substrate (Kapton), to which CNTs have low adhesion. After the solvent was evaporated, a free-standing film was obtained, from which EC was eliminated through annealing in air by igniting the film with a lighter [24].

### 2.2. Characterization

In order to determine the quality of the MWCNTs obtained, Raman spectroscopy (Renishaw, Germany) was carried out for each sample. The measurement was made using a laser with the wavelength of 514 nm, with the laser power of 50%, 20 × magnification and exposure time of 10 s. For each material, four accumulations were collected in at least four location of the sample to minimize the influence of background noise. The results are presented as averaged I_D_/I_G_ values (commonly used to gauge the purity of nanocarbon material). The value of standard deviation was determined and presented for each sample to indicate statistical significance of obtained results. 

The microstructure of the produced MWCNTs was visualized using a Scanning Electron Microscope (Supra 35; Oberkochen, Germany). SEM micrographs were obtained at 5 kV acceleration voltage. Due to the conductive nature of the CNT films, they were not sputtered with metal.

The thermal characteristic of all the samples was tested using thermogravimetry (TGA2 Mettler Toledo; Columbus, OH, USA). Measurements were performed in the temperature range between 25 °C and 1000 °C, with the heating rate of 10 °C/min and in the stream of air of 30 mL/min.

Electrical conductivity was determined using the four-probe method (Keithley 2450 SourceMeter; Cleveland, OH, USA). Thermal conductivity was probed using the steady-state method with IR thermometer (FLIR ETS 320, Wilsonville, OR, USA). Detailed description of the methodology is available in our previous publications [25].

**Figure 2:**
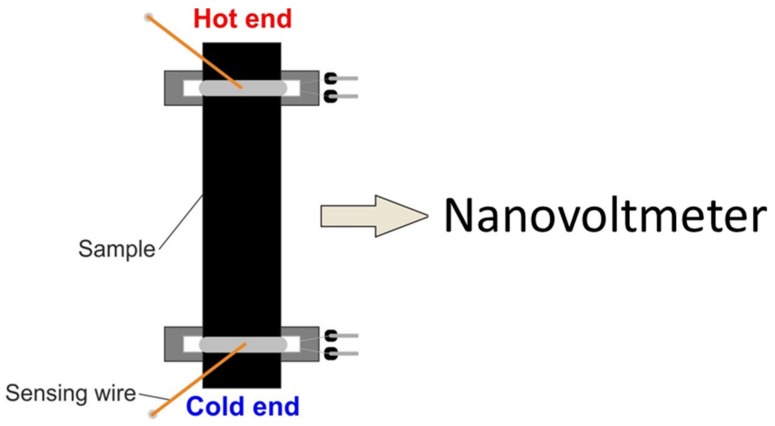
Measurement of the Seebeck coefficient of carbon nanotube (CNT) films (not to scale).

The value of the Seebeck coefficient was measured using CamSeeb 2017 (LBR, Lublin, Poland), for samples of the length of 50 mm, width of 2 mm, and thickness of approximately 0.2 mm. The temperature gradient of 5 °C was applied. The measurements were taken from 30 to 100 °C. At least three measurements were made for each material. The values were averaged, and the statistical error was determined. Scheme of the employed technique is presented in Figure 2. 

Power Factors and Figures of Merit were calculated using Equations (1) and (2) using the measured values of Seebeck coefficient and thermal/electrical conductivity recorded at 30 °C in the aforementioned way.

## 3. Results

**Figure 3:**
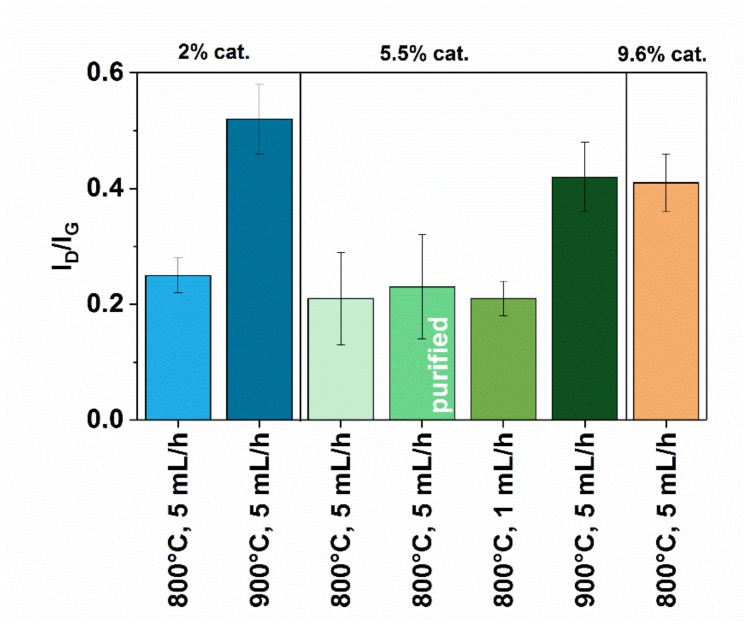
I_D_/I_G_ values of MWCNTs as determined by Raman spectroscopy.

Because conditions of synthesis often have a strong impact on the quality of obtained CNTs, we first examined the material using Raman spectroscopy (Figure 3). Generally, the samples revealed moderate purity as gauged by the ratio of intensity of defect-induced D mode and that of the band of graphitic vibrations G. For the material synthesized at 900 °C, the I_D_/I_G_ values are higher (0.52 ± 0.06) than when the reaction was conducted at 800 °C (0.25 ± 0.03). Increase of temperature in the CVD process generally leads to higher amounts of defects in the material 2%-5 mL/h-900 C because side-reactions become much apparent under such conditions. Furthermore, we wanted to explore how increase in the amount of injected catalyst would affect the composition of the CNTs. Although the increase of introduced iron as ferrocene from 2% to 5.5% does not show a notable difference, this can be ascribed to a relatively large error. Nevertheless, further increase of the amount of catalyst up to 9.6% gives elevated the level of disorder to a large extent (0.41 ± 0.05). Furthermore, purification by HCl treatment meant to remove residual catalyst as expected did not affect the chemical composition of MWCNT material because of its non-oxidizing nature (0.23 ± 0.09). Finally, changes in the injection speed did not play a role in this case.

**Figure 4:**
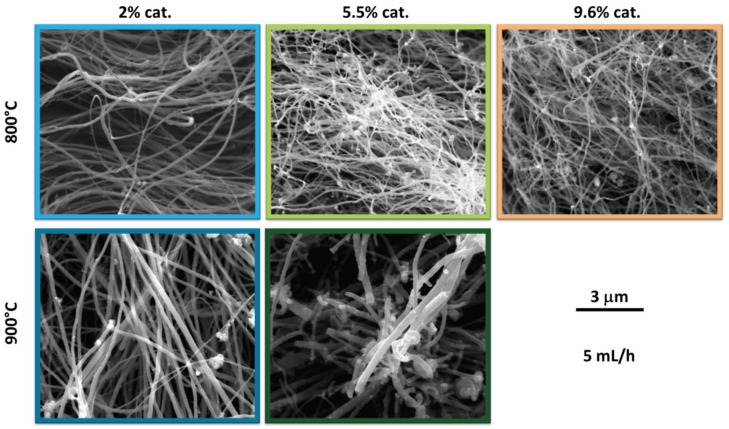
Scanning electron microscopy (SEM) micrographs of CNTs obtained at 800 °C (first row) or 900 °C (second row) with different amount of catalyst: 2% cat. (first column), 5.5% cat. (second column), or 9.6% (third column).

To gain a more detailed information about the impact of synthesis of parameters on the material we analyzed its microstructure by scanning electron microscopy (SEM) (Figure 4). 

The first evident difference is that CNTs obtained at higher temperature are characterized by significantly larger diameters, which is coherent with earlier results published by the community [26,27]. Furthermore, the second observation is that, with the increase in catalyst concentration, the MWCNTs become more intertwined and lose order. The CVD process that we employed commonly gives rise to production of aligned arrays [22] under appropriate conditions. In our case, we can see that there is some anisotropy for the sample synthesized with 2% of catalyst, but as its content is increased, the samples become fully isotropic. The material with the least appealing microstructure is the one synthesized at high temperature of 900 °C with an elevated amount of catalyst of 5.5%. Poor crystallinity and marked presence of contamination is in accordance with high I_D_/I_G_ ratio for this sample presented above. 

**Figure 5:**
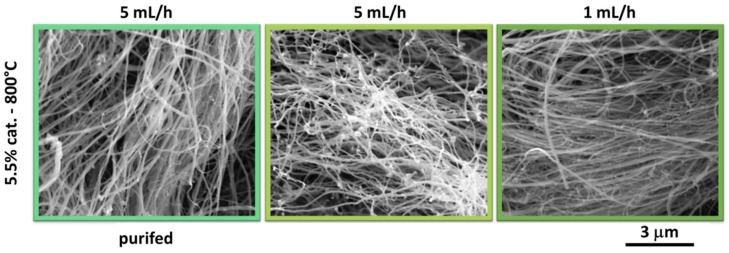
SEM micrographs of MWCNTs obtained at 800 °C and with 5.5% cat. Material purified by acid treatment has been shown on the left. Moreover, the impact of lowered injection rate (from 5 mL/h to 1mL/h) of precursor mixture has been given on the right.

We then proceeded to analyze the impact of post-synthetic purification and precursor injection rate on the microstructure of the material (Figure 5). 

In the former case, the amount of catalyst inclusions in the network has been significantly reduced (represented by bright areas in the micrographs due to their non-conducting nature). In the latter case, we see a two-fold influence. First, the amount of residual catalyst is very low, so this content of ferrocene seems closer to the optimum. Second, the material is much less tangled, and the CNTs give the impression of being longer. Since there are fewer reactive moieties in the furnace at any given moment of the synthesis, the growth appears to proceed in a controllable way along the CNT axes. This is also why the anisotropy of the material is highest among all the samples analyzed. 

**Figure 6:**
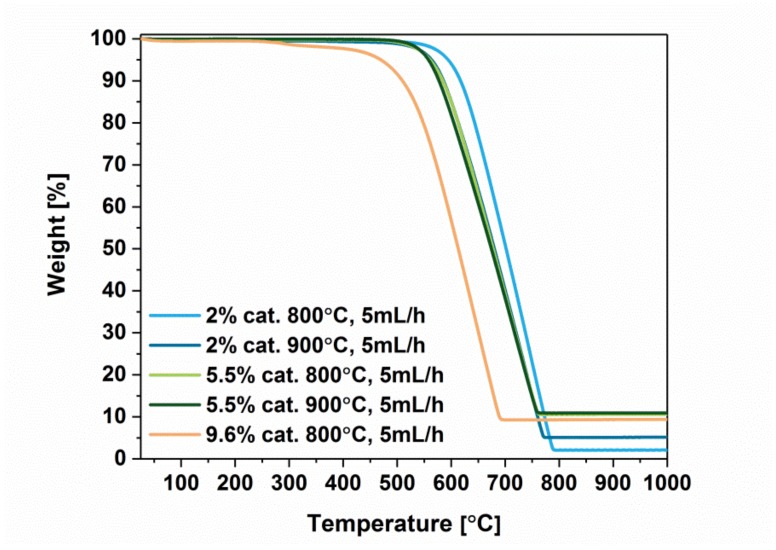
Comparison of thermograms of MWCNTs obtained at 800 °C or 900 °C with different amounts of catalyst employed (2%, 5.5%, and 9.6%).

**Figure 7:**
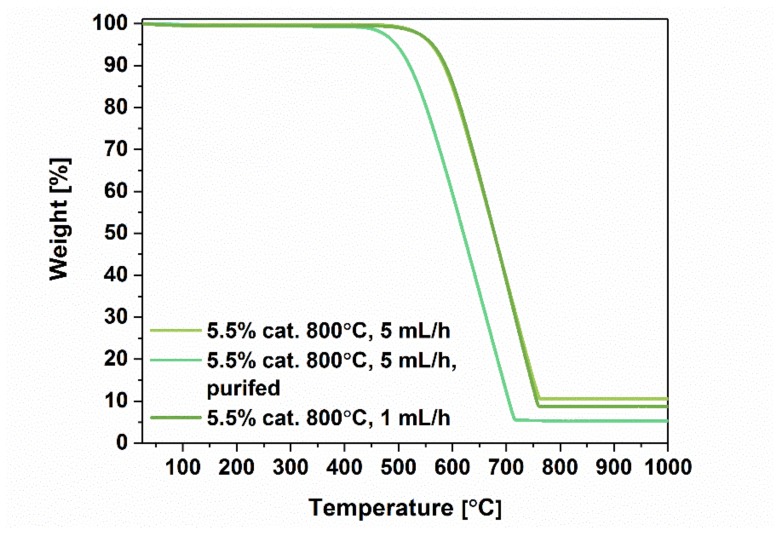
Comparison of thermograms of MWCNTs obtained with different injection speed and after purification.

To get more insight into the materials characteristics, we characterized the samples by thermogravimetry (Figure 6 and Figure 7). 

In general, MWCNTs were stable up to approximately 450–500 °C, which is consistent with data for this type of nanocarbon reported previously in the literature [28]. The lowest thermal stability is demonstrated by the material obtained with the highest amount of catalyst (9.6%, 800 °C), for which the oxidation starts already at about 450 °C. These findings are coherent with previous findings, which demonstrated that there is an abundance of defects in this sort of CNTS (confirmed by Raman spectroscopy and electron microscopy). On the other hand, highest thermal stability was recorded for material with the least amount of catalyst (2%, 800 °C) used for the synthesis. For this sample, combustion begins at almost 100 °C higher temperature (approximately 540 °C) as compared with the least stable sample. The remaining MWCNTs lose thermal stability at a similar temperature of about 500 °C. Slight differences between them may be attributed not only to a different amount of defects but also to the varying packing degree, which may often have a significant influence on the shape of the recorded thermograms [29].

Subsequently, we wanted to validate whether any difference to thermal stability can be ascribed to post-synthetic purification and change of injection speed during the synthesis (Figure 7). First, purification by acid treatment to remove residual catalyst was successful. The remaining amount in the crucible after the process constituted only 5.2% of the starting weight as compared with 10.3% for the raw material. It has to be kept in mind that some residual catalyst remains inaccessible and cannot be eliminated by such means. That is why some of the catalyst is still present in the final material. Interestingly, we observed a slight deterioration of thermal stability of the material, which decreased from 520 °C to 450 °C. Again, this could be attributed to a change in microstructure of the material caused by the processing. Packing degree can significantly affect the course of oxidation, so it is often recommended to conduct thermal analysis with a small rate of temperature increase [29]. Second, almost no difference could be ascribed when comparing thermograms of MWCNTs synthesized at 800 °C with 1 mL/h and 5 mL/h injection speed. When we refer to the values of I_D_/I_G_ for both the samples obtained from Raman spectroscopy, we can conclude that they were also very similar. Despite the fact that the material appears more ordered when synthesized at lower injection speed (Figure 5), its thermal stability remains at the same level.

**Figure 8:**
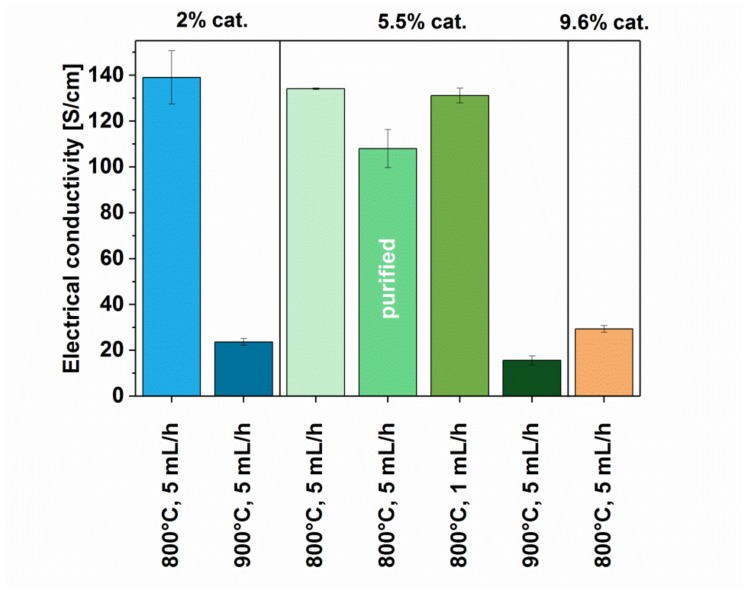
Electrical conductivity of MWCNTs synthesized using different parameters (with and without post-processing).

Next, we moved on to the characterization of the electrical, thermal, and thermoelectric properties of the networks constructed from these MWCNTs. The value of electrical conductivity was measured for the obtained MWCNT films (Figure 8), which demonstrated the factors that affect it the most are the synthesis temperature and the amount of catalyst. The highest value of electrical conductivity, i.e., 139 ± 11.6 S/cm, was recorded for MWCNTs with 2% catalyst at the temperature of 800 °C. However, when the same reagent ratios were used, but the temperature was increased to 900 °C electrical conductivity was decreased by almost an order of magnitude (23.6 ± 1.5 S/cm). Similar behavior was observed for samples synthesized with 5.5% catalyst for which a sudden drop in electrical conductivity was noted (from 134 ± 0.5 S/cm to 15.6 ± 1.9 S/cm) when we increased the temperature from 800 °C to 900 °C. As demonstrated earlier, synthesis temperature has a significant impact on the crystallographic structure of the obtained MWCNTs.

In the case of syntheses conducted at a temperature of 800 °C, it was observed that the increase in the amount of catalyst also affected the value of electrical conductivity beyond a certain threshold. Although that in the case of increasing the amount of catalyst from 2% to 5.5%, the decrease in value is insignificant (down to 134 ± 0.5 S/cm from 139 ± 11.6 S/cm), the application of 9.6% catalyst strongly deteriorates the electrical properties of the material. For this sample, electrical conductivity is decreased by about 3.5 times (eventually reaching 29.3 ± 1.4 S/cm). Furthermore, elimination of catalyst from MWCNTs has a somewhat negative impact on electrical conductivity of the material, which was reduced to 108 ± 8.3 S/cm. One of the biggest challenges to improve electrical conductivity of CNT networks is to alleviate the problem of contact resistance. Our results show that upon iron elimination we may reduce the degree of electrical percolation of the material, and thus the material becomes less conductive. Finally, we observed that changing the injection speed of precursor did not affect the electrical conductivity of the material. 

**Figure 9:**
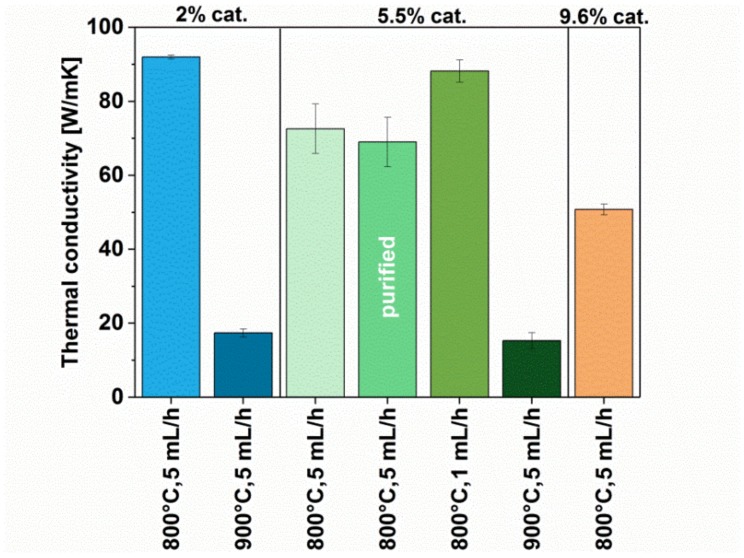
Thermal conductivity of MWCNTs synthesized using different parameters (with and without post-processing).

Results from thermal conductivity measurements (Figure 9) showed the change trends resulting from application of various synthesis parameters generally matching those observed for electrical conductivity. This is not surprising, given that both of these properties are commonly intertwined by the Wiedemann–Franz law.

By analogy to the study of electrical conductivity, the highest value of thermal conductivity was recorded for the sample obtained with 2% catalyst at the temperature of 800 °C (92 ± 0.5 W/mK). The only deviation that we noticed was for MWCNTs synthesized at 800 °C with low injection speed of precursor mixture (1 mL/h). As observed by electron microscopy, the material produced under these conditions is predominantly composed of long aligned MWCNTs. Good propagation of phonons requires appropriate contact between MWCNTs, which build up the network. It seems that, in this case, slowing down the speed of the reaction is beneficial for ensuring good thermal properties of the material. Nevertheless, the observed increase in thermal conductivity is rather small (from 72.6 ± 6.7 to 88.2 ± 3 W/mK), so this should be considered with caution. 

**Figure 10:**
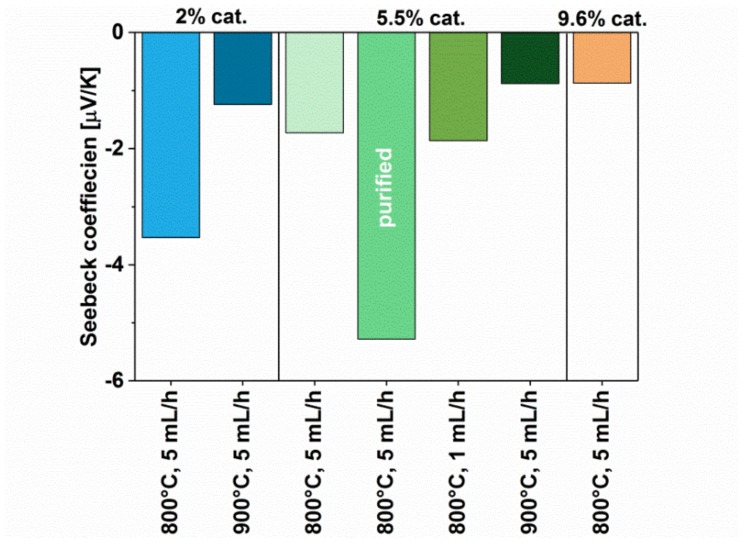
Seebeck coefficient of MWCNTs synthesized using different parameters (with and without post-processing).

For the material to be promising for thermoelectric applications, it must also possess a high Seebeck coefficient. We measured its value for all the materials and presented them in Figure 10. To our surprise, the highest value of the Seebeck coefficient was demonstrated by the material after purification (5.5%-5 mL/h-800 C-pure). It is a strong indication that residual catalyst commonly present in the material must be removed. We hypothesize that the treatment (reflux in HCl) increases the semiconducting character of the network (in principle, semiconductors have much higher Seebeck values [30]). This suspicion is in accordance with the results of electrical conductivity for this sample, which was lower than for the parent material. 

As for other materials, it was observed that the quality of crystallographic structure affects the value of the Seebeck coefficient as well. The MWCNTs made using 2% catalyst at the temperature of 800 °C are characterized by a higher absolute value of the Seebeck coefficient than the MWCNTs obtained with 5.5% or 9.6% of the catalyst. Furthermore, samples synthesized at 900 °C also had inferior Seebeck coefficients as compared to corresponding ones obtained at 800 °C. With the exception of the purified sample, the impact of the microstructure and chemical composition of the material on the Seebeck coefficient follows the influence of these characteristics on the electrical conductivity discussed above. Similar to that case, we did not detect a relation between injection speed and the value of Seebeck coefficient of the material.

**Figure 11:**
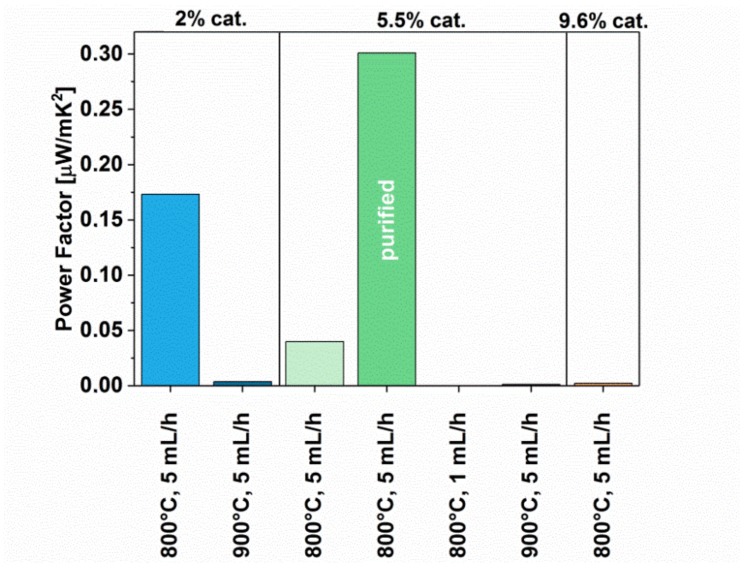
Power Factor of MWCNTs synthesized using different parameters (with and without post-processing).

**Figure 12:**
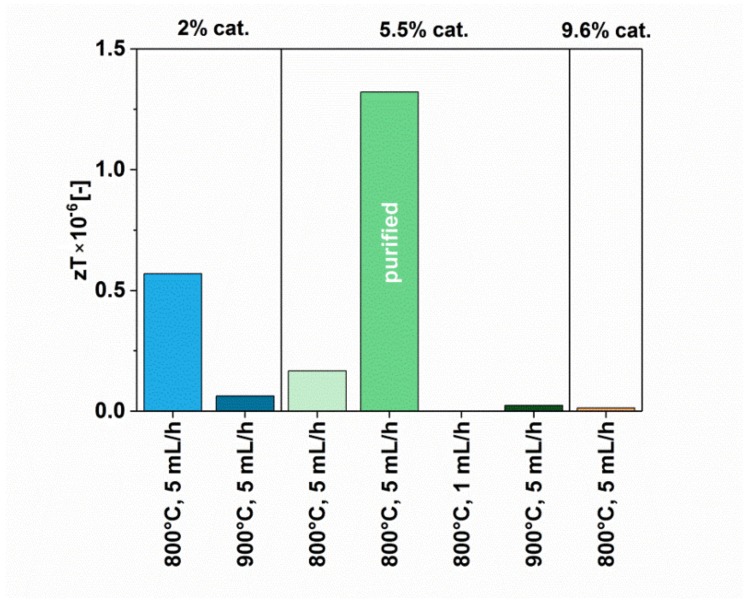
Figure of Merit of MWCNTs synthesized using different parameters (with and without post-processing).

The values that provide key information on the suitability (or unsuitability) of a material for thermoelectric applications are Power Factor (calculated based on the Seebeck coefficient and electrical conductivity, Equation (1)) and the Figure of Merit (additionally taking into the account the thermal conductivity, Equation (2)). Both these values were calculated for our materials, and the results were presented in Figure 11 and Figure 12, respectively. 

The highest value of the Power Factor of 0.3 µW/mK and zT 1.32 × 10^−6^ was demonstrated by the purified CNT film (5.5%-5 mL/h-800 C-pure), which matches our findings from determination of Seebeck coefficient. This results from the fact that the sample demonstrated the highest absolute value of the Seebeck coefficient, and relatively one of the highest values of electrical conductivity. The second-best specimen, which gave a relatively high Power Factor (0.17 µW/mK) and Figure of Merit (0.57 × 10^−6^), was for the material synthesized using reduced amount of the catalyst at 800 °C (2%-5 mL/h-800 C). That film was developed from the MWCNTs characterized by a low amount of defects, which underlines that structural perfection of the CNT building blocks is also important. However, as demonstrated by our study, the most important aspect is the nanocarbon purity of the material. The lower the content of metal nanoparticles, the better the thermoelectric performance. 

## 4. Conclusions

The conducted research demonstrated that each synthesis parameter has an influence on thermoelectric properties of produced MWCNTs. Thermal and electrical conductivity are most affected by the synthesis temperature applied or the content of the catalyst in the synthesis mixture. For instance, it has been observed that, regardless of the other parameters, the materials obtained at the temperature of 800 °C have much better heat and electrical charge propagation than when MWCNTs are synthesized at 900 °C. Nevertheless, the microstructure of the network and the degree to which the sp^2^ carbon lattice of the constituting MWCNTs is pristine enable us to predict the thermoelectric performance only to a limited extent. Despite the fact that the differences between I_D_/I_G_ ratios and topology of the samples were not drastic, some of the samples have shown much more promising thermoelectric character than anticipated. The impact on the overall thermoelectric performance is multidimensional, so further studies should be carried out to unravel the mechanics of thermoelectrics in the field of nanocarbon

Most important findings came from studying the impact of the residual catalyst. As expected, we observed that material purification reduces electrical conductivity, because iron particles, which are good conductors, are eliminated. However, removal of these unwanted species led to a significant improvement of their thermoelectric properties. This is also supported with the results from material synthesized at reduced amount of catalyst of 2%, which showed second best performance on this front. We can conclude that amount of the catalyst should be kept to the minimum to ensure good thermoelectric properties. Either the material must undergo post-synthetic purification, or the content of catalyst should already be minimized at the synthesis step to avoid further processing of the material. Outside the context of thermoelectrics, our work stresses how important it is to conduct the purification of nanocarbon before using it for R&D. The obtained results may be more encouraging, and the science done this way is also significantly more accurate and reveals the true nature of carbon nanostructures. 

Perspectives of MWCNTs for thermoelectrics are lower than that of SWCNTs because of the lack of the ability to make them fully semiconducting, which would guarantee high Seebeck effect. Nevertheless, they constitute a very convenient platform for studying the effect of their microstructure and chemical composition on the properties of the obtained materials. Hence, more importance should be put to the trends discovered by us rather than the measured absolute values. Carrying out such a proof of concept studies is vital because based on them we are able to predict the characteristics of an ideal single-walled CNT for thermoelectrics to bring them closer to real-life applications. It cannot be stressed enough that the development of such materials is of the utmost importance for the optimization of global energy consumption through energy harvesting. Better protection of the environment caused this way would be beneficial for all of us.

## Figures and Tables

**Table 1:** Various synthesis parameters to obtain MWCNTs.

Sample	Catalyst [%]	Temperature [°C]	Injection Speed [mL/h]
**2%-5 mL/h-800 C**	2%	800	5
**2%-5 mL/h-900 C**		900	
**5.5%-5 mL/h-800 C**	5.5%	800	
**5.5%-5 mL/h-800 C-pure**			
**5.5%-1 mL/h-800 C**			
**5.5%-5 mL/h-900 C**		900	
**9.6%-5 mL/h-800 C**	9.6%	800	
